# Are you PEPped and PrEPped for travel? Risk mitigation of HIV infection for travelers

**DOI:** 10.1186/s40794-016-0042-9

**Published:** 2016-11-28

**Authors:** D. M. Brett-Major, P. T. Scott, T. A. Crowell, C. S. Polyak, K. Modjarrad, M. L. Robb, D. L. Blazes

**Affiliations:** 1 Henry M. Jackson Foundation for the Advancement of Military Medicine, Bethesda, MD USA; 2 Military HIV Research Program, Walter Reed Army Institute of Research, Silver Spring, MD USA; 3 Division of Tropical Public Health, Department of Preventive Medicine and Biostatistics, F. Edward Hébert School of Medicine, Uniformed Services University, Bethesda, MD USA; 4 Bill and Melinda Gates Foundation, Seattle, WA USA

**Keywords:** Pre-exposure, Post-exposure, Prophylaxis, Travel, Human Immunodeficiency Virus, Review

## Abstract

The HIV pandemic persists globally and travelers are at risk for infection by the Human Immunodeficiency Virus (HIV). While HIV-focused guidelines delineate risk stratification and mitigation strategies for people in their home communities, travel issues are not addressed. In this review, direct and indirect evidence on HIV risk among travelers is explored. The burgeoning practice of employing pre-exposure prophylaxis (PrEP) with anti-retroviral therapy in the non-travel setting is introduced, as well as the more established use of post-exposure prophylaxis (PEP). Challenges in applying these lessons to travelers are discussed, and a new guidelines process is scoped and recommended.

## Background

Pre-exposure prophylaxis (PrEP) against the Human Immunodeficiency Virus (HIV) has been recommended in several guidelines for persons at risk within their home communities [1–3]. However, these guidelines have ignored the use of PrEP in travelers, despite the high frequency of travel both within the US and to even more HIV endemic areas. According to a travel trade association, in 2015, U.S. residents spent nearly 2.2 billion person-days traveling in the US more than 50 miles from their homes and using paid lodging [4]. In that same year, more than 350,000 U.S. residents went to Africa, 4.8 million to Asia, 7.7 million to the Caribbean and 12.6 million to Europe [5]. As this readership appreciates, travel affects behaviors and exposures, shaping risks. Here, we will explore the current options for PrEP against HIV infection and consider them in the context of travel medicine.

### What is the travel associated risk for HIV?

The GeoSentinel international surveillance network of travel clinics assessed sexually transmitted infections (STI) among its ill presenting returned travelers [6]. Among 299 men and 122 women with STI, 89 and 27, respectively, had acute HIV infection. A patient with an antiretroviral syndrome might be induced to present disproportionately to their travel medicine provider because of undifferentiated fever. Also, for pathogens like HIV which can infect people globally, GeoSentinel cannot exclude that patients contracted their STI after returning home. Nonetheless, across their cohort, STI morbidity was 6.6 per 1000 ill travelers, more than a quarter of which was HIV infection. That rate of HIV infection is nearly ten times lower than the usual universal HIV testing threshold for prevalence among presenting patients of one per cent. However, this network demonstrates that travel-associated HIV infection occurs. Passive, travel clinic case collection is just as likely to underestimate HIV infection rates as most other clinical care settings. In a large, recent study of acute HIV infection in East Africa and Thailand, patients were just as likely to not have symptoms as have them [7].

Despite awareness campaigns against supporting human trafficking through use of commercial sex, some travelers travel for sex [8]. Locations wildly differ in the degree to which such settings are regulated and in the health controls applied. Sexual tourism in particular presents a significant risk for HIV exposure and acquisition. Among UK-born adults diagnosed with HIV infection between 2002 and 2010 in England, Wales, and Northern Ireland, 15% were determined to have acquired infections outside of the U.K. These individuals most commonly traveled to the Thailand, the U.S., and South Africa and were more likely than those who acquired HIV infections in the U.K. to have heterosexual exposures and to have reported sexual contact with a commercial sex worker [9].

A number of studies have shown a positive association between travel and risky behaviors. Historical rates of casual sex exposure among travelers range as high as 50% [10]. In a survey of over 2,000 backpackers in Thailand, 2 in 3 were single and young—two thirds of respondents were under the age of 25—and mostly from Europe [11]. Of those who were single, more than a third reported having sex with a new partner. This was true more often for men and men were more likely to have sex with local persons. Longer length of stay was associated with more risk behavior. Condom use was variable. A survey of over 1,000 travelers from the U.K. visiting a wide variety of locales obtained similar results, though as might be expected the general travel population was older than the backpackers [12].

Travelers presenting for health advice on the cusp of or while working as emergency responders or long term stay humanitarian workers present special challenges. Given the numbers of persons who undertake such work, STI risks and outcomes may be underreported. A cross-sectional study of HIV risk behavior among over 1,200 globally distributed Peace Corps volunteers was undertaken in 1991 [13]. One in three single men and one in five single women had three or more sexual partners during their tours. A sero-survey of 864 Dutch expatriates was conducted in the mid-1990s [14]. One in four of those surveyed reported having unprotected sex with a local person but only two HIV infections were identified. A systematic review of pre-health advice for recent humanitarian workers identified high rates of unprotected sex, high alcohol use, depression, acute stress reaction and other HIV/ STI risk factors [15, 16].

A specialized population that may have travel associated sexual health risks is that of military personnel around the time of deployment. In a retrospective assessment of the U.S. Navy and Marine Corps HIV epidemic, nearly 1 in 10 HIV infected Sailors and Marines reported that an impending deployment contributed to their becoming infected with HIV [17]. In this same study, vacation as a risk factor for their having acquired HIV infection was cited by 1 in 5, and traveling for temporary assignment by 1 in 6. Peri-deployment samples captured incident cases [18]. This also was observed in an investigation of U.S. Army soldiers who had deployed to Afghanistan and Iraq [19]. However, both a subsequent study that explored HIV risk factors in the context of U.S. Air Force airmen mobility (deployments, duty station changes) and an internal soldier-airman case control study determined that having had increasing numbers of deployments was protective against HIV acquisition [20].

### How are antiretroviral medications used for prophylaxis?

#### Antiretroviral therapy (ART), or highly active antiretroviral therapy (HAART)

**Fig. 1:**
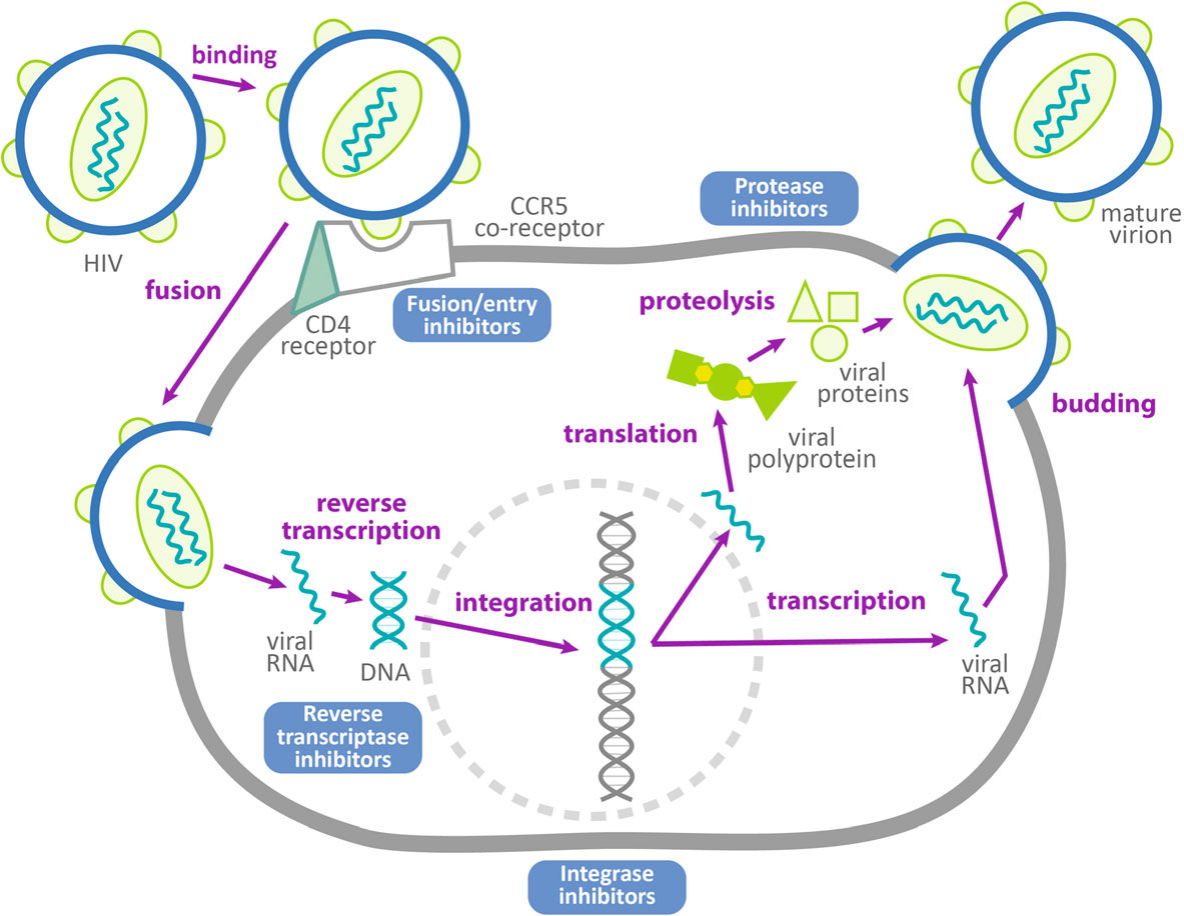
Mechanisms of anti-HIV activity by drug class

Most medications used in antiretroviral therapy against HIV infection target viral enzymes, in particular reverse transcriptase, protease or integrase. Increasingly, ART medications that block cellular receptors needed by the virus are being used. Figure 1 depicts the HIV life cycle in a cell and shows how ART drug classes apply to combating the virus. Highly active antiretroviral therapy (HAART) refers to the combination of ART medications employed together for treatment of HIV in infected persons. The nomenclature reflects the evolution of therapy from single agent in the early years of the HIV pandemic to use of three and four drug combinations as the ease with which HIV develops resistance came to be understood. Both international and country-level guidelines exist for HAART [21].

Careful review of side effects and use characteristics is important, particularly when these medications are not part of a provider’s usual practice. Comorbid conditions such as renal disease and viral hepatitis influence the choice of antiretroviral regimens. Many ART medications have significant interactions with other drugs through the induction and inhibition of metabolic pathways. Several ART:anti-malarial medication interactions are cited in the Yellow Book [22]. For instance, efavirenz decreases serum concentration of atovaquone, co-formulated with proguanil in the anti-malarial medication Malarone®. A variety of fee-based and open resources for assessing drug interaction risks also are available to help guide a provider’s choice of treatments [23–25].

### Pre-exposure prophylaxis (PrEP)

PrEP is pre-exposure prophylaxis for individuals who are not HIV infected but carry above average risk for becoming infected with HIV. The most discussed and available form of PrEP is oral antiretroviral therapy.

The first medication for which the Food and Drug Administration (FDA) approved an indication for PrEP was TRUVADA® [26]. It is a combination therapy of emtricitabine (a nucleoside analog, FTC) and tenofovir disoproxil fumarate (a nucleotide analog, TDF) that inhibits the viral enzyme reverse transcriptase. It is taken once daily as a single tablet. While the mutability of HIV and, consequently, increasing drug resistance rates have driven recommended treatment courses to triple drug therapies, recommendations for prophylaxis have remained simple and narrow. Table 1 compares the different guideline recommendations. While the guidelines do not focus on the travel clinic setting, they provide representative sexual health questionnaires and suggested criteria for both men who have sex with men (MSM) and heterosexually active men and women for determining who merits PrEP. These focus on identifying patients at increased risk because of lack of condom use, multiple sexual partners, history of sexually transmitted infections or relationships with known HIV positive partners.

**Table 1:** Current options for pharmacologic prophylaxis against HIV^a,b^

Medication	Dose (mg)	Frequency	Comments	Guidelines		
*Pre-Exposure Prophylaxis (PrEP)*				CDC	WHO	IAS
TDF + FTC	300/ 200	Daily	*TDF*- Nausea, flatulence *FTC*- rash, headache; distinguish from the treatment co-formulation with efavirenz	X	X	X
TDF	300	Daily	Not recommended alone in the US; whether in combination or alone, avoid in patients with renal injury or bone disease		X	
*non-occupational Post-Exposure Prophylaxis (nPEP)*				CDC	WHO	
TDF + FTC + RAL	300/ 200 400	Daily Twice daily	*RAL-* Mild hepatitis is common, and hypersensitivity, severe skin reactions have been reported though more common side effects include fatigue, headache, dizziness, nausea and insomnia Alternative recommendation for health adults and adolescents is TDF + FTC + DRV. In renal dysfunction, ZDV + 3TC + either RAL or DTG is recommended	X		
TDF + FTC + either LPV/r or ATV/r	300/ 200 Varies	Daily	Alternatives for the 3rd drug on the TDF + FTC backbone include RAL, DRV/r, EFV		X	
*occupational Post-Exposure Prophylaxis (oPEP)*				PHS	WHO	
TDF + FTC + RAL	300/ 200 400	Daily Twice daily	The core recommendation is to take 3 or more tolerable drugs; listed alternatives for TDF + FTC include ZDV + 3TC; listed alternatives for RAL include DRV/r, ETR, RPV, ATZ/r, LPV/r; listed alternative for all is a single co-formulation of four ART medications (TDF, FTC, EVG, cobicistat)	X		
TDF + FTC + either LPV/r or ATV/r	300/ 200 Varies	Daily	Alternatives for the 3rd drug on the TDF + FTC backbone include RAL, DRV/r, EFV WHO guidelines do not distinguish nPEP and oPEP		X	

*ATV* Atazanavir, *DRV* Darunavir, *DTG* Dolutegravir, *EVG* Elvitegravir, *ETR* Etravirine, *FTC* Emtricitabine, *3TC* Lamivudine, *LPV* Lopinivir, *RAL* Raltegravir, *RPV* Rilpivirine, *RTV* Ritonavir, /*r* boosting with ritonavir, *TDF* Tenofovir disoproxil fumarate, *TDF* + *FTC* available taken together as separate tablets or in co-formulation, *ZDV* Zidovudine

For post-exposure prophylaxis, IAS defers to CDC. The CDC produces the nPEP recommendations, while the United States Public Health Service (PHS) generated the oPEP recommendations

^a^Individual patient contraindications including drug:drug interactions, pregnancy, infections, chronic diseases such as renal or hepatic disease. They must be considered with each use. Providers should use applicable guidelines

^b^Recommendations for children differ in the guidelines with regard to age thresholds, drug selection and dosing approach

Both FTC and TDF are globally distributed and constitute the backbone of some first line HIV treatment regimens. Should contraindications to these medications exist, there are no recommended pharmacologic alternatives in the setting of pre-exposure prophylaxis. Rather, risk modifying behavior is indicated. Contraindications to TDF include renal disease (e.g., creatinine clearance below 60 mL/min), osteopenia and osteoporosis. In the United States, in single formulations for 30 tablets (one tablet per day), FTC currently costs $643.82 and TDF $1,197.32. Their co-formulation is similarly priced at $1,759.73 [27].

Research is underway for a variety of alternative PrEP strategies that use both established HIV treatment medications and other approaches. The CCR5 receptor antagonist maraviroc and the non-nucleoside reverse transcriptase inhibitor (NNRTI) dapirivine are being explored as a PrEP option, both as the sole regimen and also in combination with non-HIV medications delivered in a vaginal ring or vaginally applied tablets [28–30]. Vaginal delivery of FTC and TDF also are being assessed [31, 32]. An injectable, depot formulation of the integrase strand transfer inhibitor cabotegravir has a 40 day elimination half life. A comparison is planned between it and TDF + FTC as PrEP [33, 34]. A similar trial is in progress for a high dose, every 8 week administration of the injectable rilpirivine, a NNRTI [35, 36]. Biologic agents such as broadly neutralizing monoclonal antibodies and small molecule inhibitors also may find a niche for this indication [37].

### Post-exposure prophylaxis (PEP)

Post-exposure prophylaxis (PEP) is taken following a potential HIV exposure. Guidelines for its use are divided into two categories, occupational PEP (oPEP) and non-occupational PEP (nPEP) [38–40]. Experience with PEP is much more robust than that with PrEP. Uncertainty regarding its effectiveness under ideal circumstances as well as how long after an exposure initiating PEP still brings benefit leaves it an incomplete solution [3, 41]. Early guidelines advocated a risk tiered approach where low risk exposures employed two NRTI, higher risk exposures were an indication for the addition of a PI. Now, at least three-drug therapy is recommended (Table 1) [42]. Certain scenarios trigger a recommendation for expert consultation. These include when use is delayed, unknown sources of infection, pregnancy, breast-feeding, suspected viral resistance, toxicity of the initial regimen, serious comorbidities [42]. Suspicion or confirmation of viral hepatitis is another setting where expert consultation is prudent for PrEP, nPEP and oPEP. nPEP and oPEP are taken for 28 days.

### Who is using PrEP in their communities and to what effect?

As recommendations on use of PrEP become increasingly liberal, impact on patient and community risks is a matter for prospective study. However, success has been demonstrated in several key high-risk populations. A 2012 Cochrane review assessed four completed, randomized, controlled clinical trials that tested TDF + FTC in high-risk groups [43]. Among nearly 9,000 pooled participants, the relative risk (RR) of HIV infection for those on PrEP was 0.51 (95%CI 0.3-0.86). TDF alone also attenuated risk among 4,000 participants yielding a greater risk reduction, RR 0.38 (95%CI 0.23-0.63).

The results of practical application of PrEP in public health programs for some populations have been published. In a demonstration project on PrEP linked to sexually transmitted infection (STI) clinics in San Francisco and Miami, and a community health center in Washington DC, 557 men who have sex with men (MSM) and transgender women were followed for 48 weeks [44]. On study entry, almost 1 in 4 participants reported that their primary partner was HIV infected—typically on antiretroviral therapy—but most had additional partners. Adherence was almost 90% in San Francisco and Washington DC, but 65% in Miami. In addition to location, risk factors for poor adherence based upon dried blood spot TDF levels were being African American, and not renting or owning their own dwelling. Those with 2 or more episodes of unprotected receptive anal intercourse in the previous 3 months had higher rates of adherence. STI prevalence was high at time of screening, declined at 24 weeks but then increased again by 48 weeks. Nonetheless, only 2 HIV incident infections were observed. As these communities were selected because of an annual HIV incidence of 2% or higher, this suggests a substantial protective effect despite inevitable enrollment biases. Whether that protection is attributable only to reductions of risk at the individual level, or the presence of a collective effect because of decreased transmission within social networks is not clear.

Another open label, STI clinic-linked PrEP implementation project across 13 sites in England randomized 544 MSM participants to immediate or 1 year deferred treatment groups [45]. As in the project by Liu et al, TDF + FTC was used. The immediate PrEP group experienced 3 incident HIV infections, in contrast to 20 in the deferred PrEP group. Of these 20, 17 had been infected prior to the onset of PrEP, and the other 3 may have been. Post-exposure prophylaxis of TDF + FTC + lopinavir, a protease inhibitor (PI), was administered to 85 participants in the deferred group, 22 of which received three or more courses. Of the 20 deferred PrEP incident cases, 6 of the patients had received post-exposure prophylaxis in 12 courses of treatment. The authors estimated a number need to treat of 13—1 year of PrEP in 13 men of this population would prevent one incident case of HIV infection. As in the U.S. PrEP implementation project, STI rates were high and unperturbed.

Trials to assess PrEP adoption and use, patient and social network protective efficacy are underway now in developing areas that have particular stigma against some high-risk groups [46]. Cohorts also exist examining use among heterosexual women in Africa [47]. Studies among commercial sex workers for PrEP have focused on willingness to adopt surveys [48, 49]. Although, some clinical care site-based cohorts such as The Combine! Study in Brazil may incorporate more commercial sex workers than had previous studies [50]. The emergence and consequences of resistant HIV associated with long term PrEP remain unclear and an area for study [51].

### Counseling the traveler on HIV risk and PrEP

The most secure methods to prevent HIV acquisition are risk modifying behaviors—abstinence, appropriate condom use, knowledge of HIV status in self and partners and clean needle use. These should be reinforced in the course of travel counseling even if opting to use PrEP. Travel clinics should routinely assess sexual health risks in their patient assessments and counseling. Travel-related risks associated with HIV infection should be elicited in the pre-travel evaluation in order to identify anticipated exposures and risks associated with travel in addition to baseline, non-travel risk. For those who might benefit from long term PrEP use, the travel medicine visit should be used as an opportunity to encourage patients to discuss PrEP with their primary physician. For those with short-term risks, consider provision of non-occupational post-exposure prophylaxis (nPEP) medications and instructions on their use after a high-risk, non-occupational exposure in accordance with guidelines.

Currently, consensus guidelines do not exist to guide the unique decisions about offering PrEP to travelers who are not already taking it for pre-existing risk factors in their home communities. For the long-term stay travelers (i.e., months to years) the guidelines can be used to discuss established risk profiles in the context of the impending living environment. Per country HIV prevalence rates for some at risk areas are listed in the WHO Global Health Observatory data repository [52]. For short-term stay travelers (i.e., days to weeks), their usual risk and contexts can be reassessed in order to determine if baseline PrEP use ought to be considered. Studies have not tested the pharmacodynamics, adherence and efficacy of short term PrEP. If attempting to use PrEP for travel-related risk, a traveler may have to initiate it one to two weeks prior to travel. Pre-prescription assessments of renal function, HIV and viral hepatitis status would be prudent, as well as review of the medical history for renal disease and osteopenia. Insofar as possible, current guidelines for PrEP management should be followed. Monitoring issues will exist. For long-term travelers, in country support would have to be considered. To what degree insurance would cover episodic PrEP use for travel is not clear. Ideally, short-term travelers will incorporate their primary care providers in these discussions also as their risk may need to be reassessed.

## Conclusions and ways ahead

Key summary points are listed below.

As technologies for PrEP improve, its application in the traveler may become simpler. Depot injections that can be applied regardless of baseline health status, for instance, would be welcome. Regardless, PrEP use in the traveler, as PrEP use in communities, carries management and policy issues that merit attention. Travel and HIV interested professional societies and bodies should consider convening a dedicated review and guidelines process for this issue. Some travelers that may not be good candidates for long term PrEP could benefit from episodic, short-term PrEP associated with travel, should safe and effective approaches for this use be agreed.

Should such a guidelines effort be undertaken, several considerations useful to travel medicine providers require focused attention. These include PrEP evaluation for eligibility, minimum screening labs, PrEP initiation, toxicity monitoring, discontinuation/transition to nPEP, HIV epidemiology and resistance, and impacts upon risk reduction counseling. Both the way level of HIV risk is addressed in PrEP guidelines and the potential different sexual health risks present among varying types of travel and traveler also need to be considered. Travel medicine services are provided in a variety of settings and by a range of providers. This includes primary care providers, infectious diseases specialists, stand-alone travel medicine clinics and comprehensive travel medicine clinics integrated in infectious diseases and preventive medicine clinics. Knowledge regarding PrEP is associated with clinical experience using PrEP and ART prescribing experience [53–55]. Ideally, patients present 4–6 weeks prior to travel for evaluation by a travel medicine provider. However, patients often present much more proximal to anticipated departure and require expedited evaluation, couseling, and interventions. The lead time required to achieve protective drug levels in serum, rectal, and vaginal tissues is not entirely known. Limited existing pharmacokinetic data suggest that 7 and 20 days are required for protection at rectal tissue and cervico-vaginal tissues, respectively [56–59]. Thus, significant challenges are present in managing and counseling patients about risk and risk mitigation strategies depending on specific travel related sexual risk exposures such as insertive penile sex and receptive vaginal and anal sexual exposures and the incomplete knowledge of the estimated time to achieve protection in relevant tissues. Specific practical guidance for the travel medicine provider should include recommendations for consideration of PrEP initiation for last minute travelers with imminent travel and for transitioning travelers from PrEP to nPEP for those travelers in whom pre-travel PrEP duration may have been inadequate to achieve maximum protection. Additionally, some travelers may be healthcare workers for who occupational post-exposure prophylaxis (oPEP) needs may be present. These considerations add to the need for and complexity of a guidelines process.
